# Artificial intelligence research strategy of the United States: critical assessment and policy recommendations

**DOI:** 10.3389/fdata.2023.1206139

**Published:** 2023-08-07

**Authors:** Furkan Gursoy, Ioannis A. Kakadiaris

**Affiliations:** Computational Biomedicine Lab, University of Houston, Houston, TX, United States

**Keywords:** artificial intelligence, research, development, policy, strategy, accountable AI

## Abstract

The foundations of Artificial Intelligence (AI), a field whose applications are of great use and concern for society, can be traced back to the early years of the second half of the 20th century. Since then, the field has seen increased research output and funding cycles followed by setbacks. The new millennium has seen unprecedented interest in AI progress and expectations with significant financial investments from the public and private sectors. However, the continual acceleration of AI capabilities and real-world applications is not guaranteed. Mainly, accountability of AI systems in the context of the interplay between AI and the broader society is essential for adopting AI systems via the trust placed in them. Continual progress in AI research and development (R&D) can help tackle humanity's most significant challenges to improve social good. The authors of this paper suggest that the careful design of forward-looking research policies serves a crucial function in avoiding potential future setbacks in AI research, development, and use. The United States (US) has kept its leading role in R&D, mainly shaping the global trends in the field. Accordingly, this paper presents a critical assessment of the US National AI R&D Strategic Plan and prescribes six recommendations to improve future research strategies in the US and around the globe.

## 1. Introduction

The roots of Artificial Intelligence (AI) as a research field are usually traced back to a workshop held in 1956 on the campus of Dartmouth College (McCarthy et al., 2006). By the time of the workshop, some original ideas that characterize AI were already there. Some notable examples are Turing's seminal paper on computing machinery and intelligence (Turing, 1950), the program called Logic Theorist that could prove mathematical theorems using symbolic logic (Newell and Simon, 1956), the first neural net machine in 1951 (Crevier, 1993), and early efforts for self-learning checkers player (Sammut and Webb, 2010). As the Dartmouth workshop unified AI as a discipline, funding started to flow into AI research. However, the AI researchers overpromised, and the challenges were underestimated. Eventually, the promises were undelivered. As funders became unhappy with the progress, the amount and flexibility of funding considerably declined in the 1970s (Crevier, 1993). The following years are considered the setback years or the first AI Winter.

In the 1980s, there was a renewed interest in AI with the advent of expert systems (Crevier, 1993). Outside the United States (US) and the United Kingdom, Japan began to invest in the field (Shapiro, 1983). This period saw a great interest in knowledge representation and the revival of the interest in neural networks (McCorduck, 2004). The period is also characterized by dramatically increasing commercial interest. However, commercial vendors failed to develop workable solutions for real-world problems. The late 1980s and early 1990s also see hundreds of AI companies shutting down and the funding for AI dramatically decreasing once again (Newquist, 1994). The late 1980s and early 1990s are popularly known as the AI Winter or the second AI Winter.

AI research was reinvigorated in the late 1990s and accelerated during the new millennium. Recent years have seen a dramatic increase in the funding for AI research and commercial ventures (Mousavizadeh et al., 2021; NSF, 2021). On the other hand, some prominent researchers argue that AI abilities were overestimated in the 2010s, and they anticipate that an AI autumn might be imminent (Shead, 2020). One way to avoid such potential setbacks in AI's progress is the careful and visionary design of research policies. The National Artificial Intelligence Research and Development Strategic Plan (National Science and Technology Council, 2016), referred to as the Plan in the rest of this paper, is the current document highlighting the critical priorities for the US federal investments in AI research and development. Considering the leading role of the US, with more than 600 billion dollars in gross domestic spending on R&D in 2019 (OECD, 2022), this paper argues that the Plan has broader effects beyond the US in shaping the future of AI research. Therefore, it is worthy of a critical assessment by the academic community.

National Science and Technology Council, through which the executive branch of the US federal government coordinates science and technology policy, published the first version of the Plan in 2016 (National Science and Technology Council, 2016). Updated in 2019, the Plan (National Science and Technology Council, 2019) establishes federally funded AI research objectives by identifying eight strategic priorities. The Plan focuses on issues the industry is unlikely to handle on its own, presenting areas where federal investment is most likely to benefit.

While there are favorable views regarding the social good that AI can provide (Taddeo and Floridi, 2018; Tomasev et al., 2020), there are also studies that criticize the unjustified and hurried optimism regarding AI for social good (Moore, 2019) as well as studies that highlight the potential risks of AI (Cave and Heigeartaigh, 2018; Tzimas, 2021). Accordingly, the main contribution of this paper is to provide a critical assessment of the Plan and present recommendations to enhance the Plan toward achieving a trustworthy and safe AI that is welcome in society to progress the world toward a techno-social paradigm. In this way, humans and accountable AI systems can collaborate to address society's most significant challenges, keeping the social good and progress at the center. The remainder of this paper is structured as follows. Section 2 provides summary descriptions of the eight strategic priorities. Section 3 presents and discusses recommendations to strengthen the Plan. Final remarks and conclusions are provided in Section 4.

## 2. Strategic priorities

The Plan outlines eight strategies. The strategies span the entire field rather than responding to or highlighting individual research challenges. The first and second strategies include R&D areas where further progress is needed to advance AI. The remaining six strategies are presented as the cross-cutting R&D foundations affecting the development of AI systems. Based on these eight strategic priorities, future enhancements in the field of AI are expected to assist individual applications of AI. Next, we review and briefly explain each strategy.

The first strategy is concerned with making long-term investments in AI research. In addition to the incremental research with predictable short-term outcomes, this strategy aims to sustain long-term research that may be riskier but potentially have very large payoffs. The strategy explicitly highlights (i) knowledge discovery from multi-modal, noisy, and big data; (ii) perceptual capabilities of AI systems via sensors and other means; (iii) understanding of theoretical limitations of AI concerning available hardware; (iv) general-purpose artificial intelligence that is capable of performing different kinds of tasks like humans do; (v) coordination of multi-AI systems; (vi) human-like AI that can learn from small sample sizes, and that can explain itself; (vii) robotic technologies; (viii) hardware specialized for AI; and (ix) AI for improving hardware design. This strategy mentions several vital concepts such as perception and attention, commonsense and probabilistic reasoning, combinatorial optimization, knowledge representation, natural language processing, and human-machine interaction as prioritized areas for fundamental AI research.

The second strategy is concerned with developing effective methods for human-AI collaboration. The strategy suggests that many applications of AI will not be completely autonomous. Instead, a combination of AI and human systems will work together. An effective and efficient human-AI collaboration requires additional R&D. The strategy highlights some development challenges: (i) human-aware intelligent systems that are capable of intuitive interaction with humans; (ii) AI techniques that enhance human capabilities, for instance, through wearable devices; (iii) human-AI interfaces to present increasingly complex data in a human-understandable manner; and (iv) better language processing systems that overcome current challenges such as noisy surroundings, heavily accented speech, impaired speech, and real-time dialogue with humans. The strategy also argues that trust in AI is necessary for human-AI collaborations, which is related to fairness, explainability, and transparency.

The third strategy is concerned with understanding and addressing AI's ethical, legal, and societal implications. This strategy focuses on fundamental concepts such as trustworthiness, fairness, transparency, accountability, explainability, and ethics. The strategy presents three subsections to explore critical challenges: (i) incorporating fairness, transparency, and accountability in the design of AI systems, (ii) building ethical AI; and (iii) designing system architectures incorporating ethical reasoning.

The fourth strategy is concerned with ensuring the safety and security of AI systems. The strategy emphasizes the vital role of safety and security in achieving robust and trustworthy AI systems. The strategy presents several challenges: (i) developing AI systems that are capable of explaining the reasons behind the outputs they produce; (ii) building trust in AI; (iii) enhancing verification and validation of AI systems by meeting formal specifications and user's operational needs, respectively; (iv) robustness against cyber-attacks; and (v) developing self-monitoring architectures for the safety of self-modifying systems.

The fifth strategy involves developing shared public datasets and environments for AI training and testing. The strategy presents three critical areas of importance: (i) developing a wide variety of accessible datasets for the needs of the whole spectrum of AI applications; (ii) ensuring responsiveness of training and testing resources to public and commercial needs; and (iii) open-source software for making AI technologies more accessible. The strategy further stresses the importance of findability, accessibility, interoperability, and reusability principles for datasets and potential privacy and bias issues in datasets. Moreover, the need for computational resources to process data is underlined.

The sixth strategy is concerned with measuring and evaluating AI technologies based on well-established standards and benchmarks. The strategy highlights several areas as needing further progress: (i) developing AI standards for the broad spectrum of AI; (ii) establishing benchmarks for evaluating AI and its compliance to the standards; (iii) increasing the availability of testbeds in all areas of AI; and (iv) engaging users, industry, government, and academia in standards and benchmarks. Further, the strategy calls attention to measuring and evaluating AI systems to assess and assure safety, security, privacy, robustness, explainability, transparency, and fairness.

The seventh strategy is to understand better the national AI R&D workforce needs. It highlights the increasing demand for AI expertise and calls for improving the existing efforts for advancing the AI R&D workforce. The strategy explicitly mentions enhancing instructional capacity from K-12 to graduate level, nurturing computer scientists and experts from other fields such as cognitive science, economics, linguistics, and others.

The last strategy concerns expanding public-private partnerships to accelerate advances in AI. The strategy explicitly states government, universities, and industry entities for public-private partnerships. The benefits of such collaboration include leveraging resources to push innovation, supporting the practices based on these innovations, and enhancing the training for future researchers and practitioners.

## 3. Recommendations

The increasingly decisive role of AI in people's lives necessitates a sociotechnical viewpoint (Sartori and Theodorou, 2022) that encompasses everything from the conception of an AI system to the consequences of its use in the real world. Such a sociotechnical viewpoint concerns interactions and other complex relations between human and AI systems (Herrmann and Pfeiffer, 2022). The current version of the National Artificial Intelligence Research and Development Strategic Plan already addresses several sociotechnical aspects. This section proposes and discusses six recommendations to enhance the Plan for achieving trustworthy AI.

I. The first strategy describes fundamental AI research areas where further efforts are encouraged. While the topics around Causal AI (Yao et al., 2021; Scholkopf, 2022) are already receiving increasing attention from the machine learning community, the Plan does not discuss causality in AI. However, it is still a domain with challenging questions and potentially significant benefits (Dhar, 2020). Exploring causal relations in a system helps us understand the system and potentially improve AI applications (Sgaier et al., 2020). Causal AI also provides tools for Explainable AI (Chou et al., 2022) and fairness (Mitchell et al., 2021), for instance, via counterfactual analysis (Kasirzadeh and Smart, 2021). Another key topic that is worthy of inclusion is symbolic and connectionist approaches to AI (Goel, 2022) and their potential integration, which are tightly linked with explainability of AI, learning efficiency, and knowledge representation.
*Recommendation:* The Plan should include Causal AI and the integration between symbolic and connectionist approaches as additional areas that require commitment for long-term fundamental research. Future research will help AI advance to the next stage in its capabilities, robustness, fairness, and explanatory power.
II. The second strategy addresses human-AI collaboration. However, it primarily focuses on creating “AI systems that effectively complement and augment human capabilities.” It acknowledges the challenges regarding human-aware AI, AI techniques for human augmentation, human-AI interfaces, and language processing systems. In general, these challenges are concerned with improving AI systems. However, improving human-AI collaboration does not depend solely on technical improvements regarding AI and its interfaces or mechanistic details of how humans collaborate with AI. In addition, it requires an understanding and improvement of how humans interact with and perceive the decisions or other outputs produced by AI systems (Bader and Kaiser, 2019; Araujo et al., 2020; Meissner and Keding, 2021). Human oversight of AI (Wagner, 2019; Koulu, 2020) is an area where further research is needed to understand how human decision-makers may influence or be influenced by AI decisions and to design appropriate and feasible monitoring and oversight mechanisms necessary to improve trust toward AI systems and minimize risks and harms.
*Recommendation*: The Plan should support research initiatives that tackle questions related to understanding and improving when and how humans can oversee and modify the decisions by AI systems such that the adoption of AI in relatively higher-risk situations may be increased while avoiding unacceptable risks.
III. The third strategy describes three key research challenges in AI's ethical, legal, and societal implications. These are (i) improving fairness, transparency, and accountability by design, (ii) building ethical AI, and (iii) designing architectures for ethical AI. However, as described in the Plan, these three challenges largely overlap without clear and intuitive distinctions. Also, explainability is discussed in the fourth strategy, which is concerned with the safety and security of AI systems. In contrast, this paper argues that it is more appropriate to discuss explainability concerning the other components of the third strategy and within its scope.
*Recommendation*: The third strategy may be rewritten to present notions and challenges concerning social implications and accountability of AI systems, which include concepts such as responsibility, explainability, robustness, and fairness. It should also contain references to other related strategies, such as the second strategy on human-AI collaboration, the fourth strategy on privacy and security of AI systems, and the sixth strategy on developing methods, metrics, benchmarks, and standards to evaluate AI systems.
VI. The trust to be placed in AI and its expanding role in society depends not only on the benefits of AI but also on its risks, potential harms, and remedies (Knowles and Richards, 2021). Regardless of the efforts that are possibly spent to make AI systems safe, it is not typically attainable to ensure a given AI system is perfectly safe and free from risks (Alfonseca et al., 2021). When due efforts are not provided or unknown/undiscovered factors are in play, known risks increase and unknown risks emerge. To improve trust in future AI systems, on the one hand, the types and nature of unknown and typically undiscovered risks should be explored by future research. On the other hand, remedy mechanisms should be developed and implemented. Such efforts closely relate to risk ratings, certifications, and insurance for AI. Especially given the unattainability of perfect AI systems, insurance is a helpful and necessary mechanism. However, for AI systems, evaluating the probability and severity of risks and harms is not currently feasible, which provides an obstacle for AI insurance to emerge due to the uncertainties around pricing or settlements.
*Recommendation*: The Plan should support research initiatives that tackle questions related to understanding and operationalizing the risks and harms of AI systems so that risk ratings, certifications, and insurance become feasible for AI systems. This recommendation relates to Strategies 3, 4, and 6.
V. The seventh strategy addresses the increasing demand for AI researchers and practitioners. While it acknowledges that the AI workforce is not composed only of computer and information scientists and engineers but also includes multidisciplinary teams, it appears to present the other fields and domains as areas “in which AI may be applied.” We suggest that multidisciplinary work where people from different disciplines work together is insufficient. Instead, an interdisciplinary and transdisciplinary approach (van den Besselaar, 2001) is needed to integrate knowledge from various disciplines to cross disciplinary boundaries to employ a holistic perspective. Accordingly, there is a growing need for social scientists with backgrounds in anthropology, economics, education, law, linguistics, political science, psychology, and sociology to conduct interdisciplinary and transdisciplinary research on the challenging problems at the crossroads of AI and social sciences (Kwok, 2019; Royer, 2019).
*Recommendation*: Considering the emerging intertwined nature of AI and human lives, the importance of cultivating an interdisciplinary and transdisciplinary AI workforce should be emphasized.
VI. The eighth strategy supports expanding public-private partnerships focusing on government-university-industry research and development partnerships. Given the social implications of AI, civil society organizations play a relevant and valuable role in representing the expectations of the broader society.
*Recommendation*: The eighth strategy should be expanded to include collaboration with civil society organizations, particularly concerning future developments regarding the societal implications of AI.

## 4. Conclusion

The US is leading in shaping AI research and development trends globally. Such trends are highly relevant for the future of the field, especially to direct resources to prevent another AI Winter, improve social good, and ensure the safe progress of the society toward the new sociotechnical paradigm. Given this pressing issue, this paper investigates the official AI R&D strategies of the US government with a critical lens. It offers six recommendations to improve AI research strategies in the US and beyond.

The first recommendation calls for more fundamental research on causality in AI. The second recommendation calls for a better understanding of and mechanism design for human oversight of AI. The third recommendation calls for a clear and comprehensive presentation of accountable AI to guide future research. The fourth recommendation calls for further efforts to facilitate risk ratings, certifications, and insurance for AI systems. The fifth recommendation calls for more interdisciplinary and transdisciplinary research. Finally, the sixth recommendation calls for the participation of civil society actors in AI research collaborations.

## Data availability statement

The original contributions presented in the study are included in the article/supplementary material, further inquiries can be directed to the corresponding author.

## Author contributions

FG and IK contributed to conception and design of the study, contributed to manuscript revision, read, and approved the submitted version. FG wrote the first draft of the manuscript. All authors contributed to the article and approved the submitted version.
